# Antimicrobial resistance and rational use of medicine: knowledge, perceptions, and training of clinical health professions students in Uganda

**DOI:** 10.1186/s13756-022-01186-9

**Published:** 2022-11-25

**Authors:** Andrew Marvin Kanyike, Ronald Olum, Jonathan Kajjimu, Shebah Owembabazi, Daniel Ojilong, Dianah Rhoda Nassozi, Joan Fidelia Amongin, Linda Atulinda, Kenneth Agaba, Drake Agira, Nicholas Kisaakye Wamala, Richard Buule, Germinah Nabukeera, Robert Kyomuhendo, Rehema Luwano, Whitney Owobusingye, Dissan Matovu, Philip Musoke, Felix Bongomin, Kenedy Kiyimba

**Affiliations:** 1 Faculty of Health Sciences, Busitema University, Mbale, Uganda; 2 School of Medicine, College of Health Sciences, Makerere University, Kampala, Uganda; 3 Faculty of Medicine, Mbarara University of Science and Technology, Mbarara, Uganda; 4 Faculty of Health Science, Lira University, Lira, Uganda; 5 School of Medicine, Kabale University, Kabale, Uganda; 6 Faculty of Clinical Medicine and Dentistry, Kampala International University, Ishaka-Bushenyi, Uganda; 7 Faculty of Medicine, Gulu University, Gulu, Uganda; 8 Faculty of Medicine, Islamic University in Uganda, Mbale, Uganda; 9 Faculty of Nursing and Health Sciences, Bishop Stuart University, Mbarara, Uganda; 10 Faculty of Biology, Medicine and Health, King Ceaser University, Kampala, Uganda; 11 Department of Medical Microbiology and Immunology, Faculty of Medicine, Gulu University, Gulu, Uganda; 12 Department of Pharmacology and Therapeutics, Faculty of Health Sciences, Busitema University, Mbale, Uganda

**Keywords:** Antimicrobial resistance, Rational use of medicine, Health profession students, Knowledge

## Abstract

**Background:**

Antimicrobial resistance (AMR) is an important global health concern, projected to contribute to significant mortality, particularly in developing countries. This study aimed to determine the knowledge, perceptions of clinical health professions students towards antimicrobial resistance and rational use of medicine and confidence level to prescribe antimicrobials.

**Methods:**

An online descriptive cross-sectional survey was conducted among clinical health professions students across 9 medical schools in Uganda. A semi-structured questionnaire using Kobo Toolbox form was shared among participants via WhatsApp Messenger (Meta, California, USA). Knowledge was categorized using modified Bloom’s cut-off. One-way ANOVA, Chi-square or Fisher’s exact test, and logistic regression were used to assess the association between dependent and independent variables. A *p* < 0.05 was considered statistically significant.

**Results:**

We surveyed 681 participants, most were pursuing a Bachelor of Medicine and Surgery degree (*n* = 433, 63.6%), with a mean age of 24 (standard deviation: 3.6) years. Most participants (*n* = 596, 87.5%) had sufficient knowledge about antimicrobial resistance with a mean score of 85 ± 14.2%. There was a significant difference in mean knowledge scores of year 4 (86.6%) compared to year 3 (82.4%) (*p* = 0.002) and year 5 (88.0%) compared to year 3 (82.4%) (*p*** < **0.001). Most participants (*n* = 456, 66.9%), were confident on making an accurate diagnosis of infection, and choosing the correct antimicrobial agent to use (*n* = 484, 71.1%).

**Conclusion:**

Health profession students exhibited good knowledge on antimicrobial resistance and high self-perceived confidence that should be leveraged to foster better future antimicrobial prescription practices. However, they still agreed that a separated course unit on AMR is necessary which responsible authorities should consider to consolidate the efforts.

**Supplementary Information:**

The online version contains supplementary material available at 10.1186/s13756-022-01186-9.

## Background

The discovery of penicillin by Sir Alexander Fleming in 1928 is one of the greatest revolutions in therapeutics and practice of modern medicine [1]. However, the current surge in antimicrobial misuse and overuse in human, agricultural and veterinary practices have escalated world-wide spread of antimicrobial-resistant organisms which has emerged as a big threat to global health [2–4]. In 2019 alone, an estimated 1.27 million deaths were directly attributed to bacterial antimicrobial resistance (AMR), with sub-Saharan Africa having the greatest burden [5]. It is projected that by 2050, AMR will cause up to 10 million deaths, especially in low- and middle-income Countries like Uganda which carry the greatest burden of severe and life-threatening infections [6, 7] and approximately USD 100 trillion of the world’s fiscal outputs will be splurged if definitive measures to contain the burden are not implemented [8]. Although AMR develops naturally, factors like poor prescription practices, improper medication use by patients, and in some instances, inadequate health workers’ knowledge on AMR accelerate its development [9, 10].

The World Health Organization (WHO) defines parameters for good prescription practice [11], and in collaboration with International Network of Rational Use of Drugs (INRUD), developed indicators to measure performance of rational antimicrobial prescription [12]. Prescriptions are largely done by qualified health professionals like doctors, whose shortage especially in low- and middle-income countries exacerbate irrational medicine usage [4, 13, 14]. Medical students are expected to prescribe drugs under supervision of clinical instructors as its imperative that the next generation is better prepared to use antimicrobials more sparingly and appropriately. The WHO emphasizes that healthcare workers and medical students should be trained on rational antimicrobial prescribing or antimicrobial stewardship as an integral part of efforts to curb antimicrobial resistance [3, 15]. Uganda in 2018 developed a national action plan against antimicrobial resistance entailing integration of antimicrobial stewardship courses into training curriculum for undergraduate courses though its implementation is still unsatisfactory [16].

The medical school curriculum generally provides students with theoretical knowledge of biomedical sciences, diagnosis, treatment, and prevention of diseases but does not set up antimicrobials and their practical rational prescription as a separate entity. Indeed, from a study by Huang et al. in 2013, clinical students exhibited good knowledge about antimicrobials however their usage and prescription were excessive and majority believed that it’s important to establish a separate course unit on rational use of antimicrobials [14]. Several other studies have also reported similar findings where medical students lacked self-confidence on antimicrobial prescription, believed inadequate time is spent on clinical pharmacology and would recommend more training on rational prescription [17–21].

Health professions students constitute the next-generation medical personnel whose knowledge, attitude and practice in relation to antimicrobial use can greatly impact in the future of antimicrobial resistance [14]. In Uganda, during the 5-year medical undergraduate study, students in the clinical years are expected to prescribe and manage patients under supervision. However, there is inadequate understanding of medical students’ knowledge and perceptions on antimicrobial resistance, confidence level of prescription and whether they receive adequate education on appropriate antimicrobial use. This study aimed to explore important concepts on rational medicine use among clinical health professions students in Uganda.

## Methods

### Study design

We conducted a descriptive cross-sectional, multicenter, online survey for 2 weeks within the month of October 2021 using a quantitative approach.

### Study setting

The study involved 9 Universities in Uganda offering undergraduate health science courses namely Makerere University (MAK), Mbarara University of Science and Technology (MUST), Gulu University (GU), Kampala International University (KIU), Kabale University (KU), Busitema University (BU), Islamic University in Uganda (IUIU), Lira University (LU), and Bishop Stuart University (BSU). MAK, GU, MUST, BU, LU and KU are public universities, and the remaining universities are private.

### Study population

The study involved health professions students specifically in their clinical years pursuing any of the following courses: Bachelor of Medicine and Bachelor of Surgery (MBChB), Bachelor of Dental Surgery (BDS), Bachelor of Science in Nursing (BNS), Bachelor of Pharmacy (BPharm) and Bachelors of Anesthesia (BNA). In Uganda the different Universities run different 5-year curriculums of MBChB and BDS, 4 years for BNS, BNA, and BPHARM where some students start clinical studies in the third year while others in the fourth year. Therefore, the study targeted students from year three to five in the different universities considering that they were in their clinical phase of study.

### Inclusion and exclusion criteria

Students in the above-mentioned Universities pursuing MBChB, BDS, BNS, BNA, BPharm courses in their clinical years who had consented to participate were included in the study while those who didn’t consent to participate were excluded.

### Data collection and sampling procedure

This study was conducted at a time that Uganda was still in a partial lockdown. We therefore opted to use WhatsApp Messenger (Meta, California, USA) for enrolling potential participants. We employed convenience sampling where we identified all the existing WhatsApp groups of health professions students in the nine universities through a coordinator for each specific group. The Kobo Toolbox link to the questionnaire was then sent to the potential participants via the identified WhatsApp groups.

### Data collection tool

The data collection tool was developed from validated questionnaires of similar studies [3, 4, 22, 23] and modified to suit our study objectives and locality (Additional file 1: Questionnaire.docx). It was aggregated in four parts the first one collecting participants’ demographic information. Knowledge about antimicrobial resistance was assessed using 5 questions 3 on general aspects and 2 on national AMR patterns. Each question had a value of ‘1’ for correct response or ‘0’ for wrong or don’t know response. Then it was categorized using modified Bloom’s cut-off point, as sufficient if the score was above 80%. The knowledge on rational use of medicine was assessed with 5 questions assessing awareness of different aspects with a ‘yes’ or ‘no’ response. Third part consisted of perceptions of students about antimicrobial resistance with 6 statements answered on a 5-point Likert scale from strongly agree to strongly disagree and 5 statements about perceptions on quality of the training on antibiotic resistance and rational use of medicine answered by 3-Likert scale of “yes”, “no”, or “unsure”. The last assessed Confidence level of prescription among medical Students answered on a 5-point Likert scale from Very confident to Very unconfident assigned a score of 5 through 1.

### Study variables

Independent variables were the demographic details which included sex, age, religion, residence, and University and dependent variables were knowledge, attitude, perceptions on antimicrobial resistance and rational use of medicine, perception on training and level of prescription confidence.

### Data management analysis

Fully completed questionnaires were extracted from Kobo Toolbox and exported to Microsoft Excel 2016 (Microsoft Corporation) for cleaning and coding. The cleaned data was exported to Stata (StataCorp) version 16 for analyses. Numerical data was summarized as means and standard deviations. Categorical data was summarized as frequencies and proportions. Associations between independent variables and dependent variables were assessed using one way ANOVA, Chi-square test or Fischer’s exact test and multivariate logistic regression analysis. A *P* < 0.05 is considered statistically significant.

## Results

### Sociodemographic characteristics of participants

A total of 681 participants (55% response rate), with a mean age of 24 ± 3.6 years were enrolled. Of this, most were male (*n* = 381, 55.9%), in their third year of study (*n* = 314, 46.1%), and pursuing MBChB (*n* = 433, 63.6%), Table 1.

**Table 1:** Sociodemographic characteristics and knowledge scores of participants on antimicrobial resistance (*N* = 681)

Variable	Frequency (%)	Knowledge (% Score) Mean ± SD	Sufficient knowledge		*p* value
			Yes (%)	No (%)	
Overall	85 ± 14.2	596 (87.5)	85 (12.5)		
Age (Mean ± SD)	24 ± 3.6 years				
*Sex*					
Male	381 (55.9)	85.4 ± 15	325 (54.5)	56 (65.9)	0.049*
Female	300 (44.1)	84.4 ± 13.2	271 (45.5)	29 (34.1)	
*Year of study*					
Year 3	314 (46.1)	82.4 ± 13.2	272 (45.6)	42 (49.4)	0.806
Year 4	220 (32.3)	86.6 ± 14.4	194 (32.6)	26 (30.6)	
Year 5	147 (21.6)	88.0 ± 15.3	130 21.8)	17 (20.0)	
*Course*					
Medicine & surgery	433 (63.6)	86.1 ± 15.6	365 (61.2)	68 (80.0)	0.016*
Nursing	206 (30.3)	82.3 ± 10.7	191 (32.0)	15 (17.6)	
Pharmacy	25 (3.6)	86.4 ± 11.1	24 (4.0)	1 (1.2)	
Dental surgery	9 (1.3)	91.1 ± 10.5	9 (1.5)	0 (0)	
Anesthesia	8 (1.1)	77.5 ± 16.7	7 (1.2)	1 (1.2)	
*University*					
Lira university	150 (22.0)	81.6 ± 8.8	143 (24.0)	7 (8.2)	0.014*
Busitema university	114 (16.7)	87.5 ± 15.4	100 (16.8)	14 (16.5)	
Kampala international	113 (16.6)	83 ± 15.9	92 (15.4)	21 (24.7)	
Mbarara university	76 (11.2)	85.3 ± 14.4	68 (11.4)	8 (9.4)	
Gulu university	69 (10.1)	84.6 ± 16.9	55 (9.2)	14 (16.5)	
Kabale university	63 (9.3)	86.7 ± 14.8	53 (8.9)	10 (11.8)	
Makerere university	50 (7.3)	89.2 ± 14.1	46 (7.7)	4 (4.7)	
Bishop Stuart university	26 (3.8)	83.1 ± 14.6	21 (3.5)	5 (5.9)	
Islamic university in Uganda	20 (2.9)	90 ± 13.8	18 (3.0)	2 (2.4)	
*Prior antimicrobial resistance training*					
Yes	481 (70.6)	85.3 ± 13.5	430 (72.1)	51 (60.0)	0.021*
No	200 (29.3)	84.2 ± 15.9	166 (27.9)	34 (40.0)	

*Statistically significant at* P* < 0.05

### Knowledge of participants on antimicrobial resistance and rational use of medicine

**Fig. 1:**
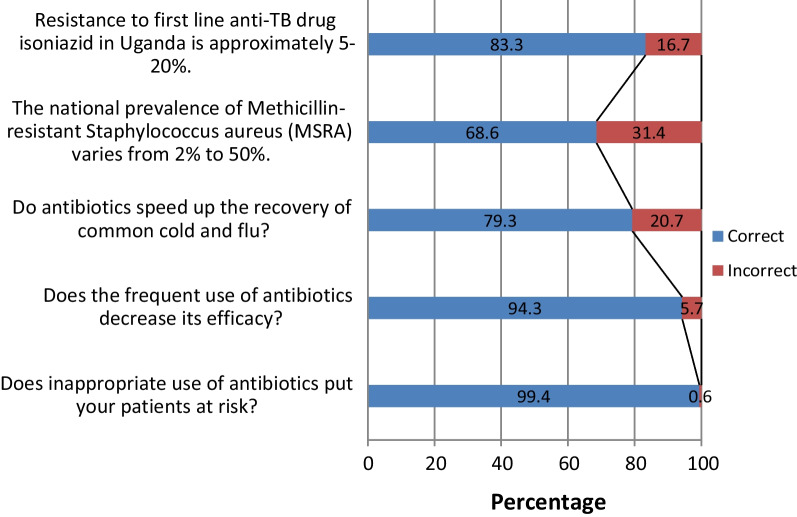
Responses participants to knowledge questions on antimicrobial resistance

Most participants (*n* = 596, 87.5%) had sufficient knowledge about AMR with a mean score of 85 ± 14.2%. A higher proportion of female than males participants (54.5% vs. 45.5%, *p* = 0.049) and those who had received a prior training on AMR than not (72.1% vs. 27.9%, *p* = 0.021) had sufficient knowledge (Table 1). Majority of students knew the fact that antibiotics don’t speed up recovery of common cold (*n* = 540, 79.3%), Fig. 1. On bivariate analysis, sex (*p* = 0.049), course (*p* = 0.016), university (*p* = 0.014) and prior training on AMR (*p* = 0.021) were significantly associated with knowledge on AMR, Table 1. However, they all lost significance at multivariate logistic regression analysis.

There was a statistically significant difference in level of knowledge on AMR across year of study (*p* < 0.001) and course (*p* = 0.006). Mean knowledge scores of fourth year students were significantly higher than those of third year (86.6% vs. 82.4%, *p* = 0.002), and fifth-year students higher than those of third year students (88.0% vs. 82.4%, *p* < 0.001). There was no significant difference between those of 4th and 5th year students (*p* = 0.622). Also, the mean score of students pursuing MBChB (86.1%) was higher than those doing Nursing (82.3%) (*p* = 0.006), Table 2.

**Table 2:** Post Hoc Tukey HSD results for comparison groups according to year of study and course

comparison group	Mean 1	Mean 2	Mean difference	95% CI of difference	*p* value
*According to year of study*					
Year 5 Vs. Year 4	88.03	86.64	1.391	− 2.125 to 4.907	0.622
Year 5 Vs. Year 3	88.03	82.36	5.679	2.372 to 8.969	< 0.001*
Year 4 Vs. Year 3	86.64	82.36	4.280	1.378 to 7.182	0.002*
*According to course of study*					
Medicine and Surgery vs. Nursing	86.14	82.33	3.813	0.8254 to 6.801	0.006*
Medicine and Surgery vs. Pharmacy	86.14	86.40	− 0.2568	− 7.518 to 7.004	> 0.999
Medicine and Surgery vs. Dental Surgery	86.14	91.11	− 4.968	− 16.86 to 6.920	0.754
Medicine and Surgery vs. Anesthesia	86.14	77.50	8.643	− 3.952 to 21.24	0.304

*Statistically significant at* P* < 0.05

**Fig. 2:**
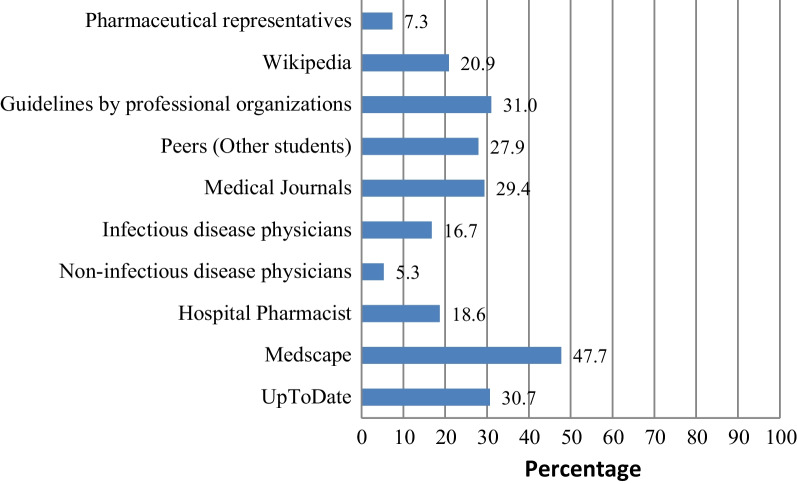
Sources of Information on antimicrobial resistance and rational drug

On rational use of medicine, most participants reported awareness of the terms rational use of medicine (*n* = 631, 92.7%), essential medicine list (*n* = 586, 86.1%) and could name parts of a prescription (*n* = 600, 88.1%), Table 3. Most participants used Medscape (47.7%) as the major source of information on AMR and rational use of medicine, Fig. 2.

**Table 3:** Responses of participants on knowledge questions on rational use of medicine

Question	Yes frequency (%)	No frequency (%)
Are you aware of the term Rational use of medicine?	631 (92.7)	50 (7.3)
Are you aware of the term essential medicines Lists (EML)?	586 (86.1)	95 (13.9)
Are you aware of the term P-drugs?	505 (74.1)	176 (25.8)
Can you name the parts of a prescription?	600 (88.1)	81 (11.9)
Are you aware of STEP (Safety, tolerability, efficacy, price) criteria for selection of P-drug?	459 (67.4)	222 (32.6)

### Perceptions of participants towards and on their quality of training on antimicrobial resistance

**Fig. 3:**
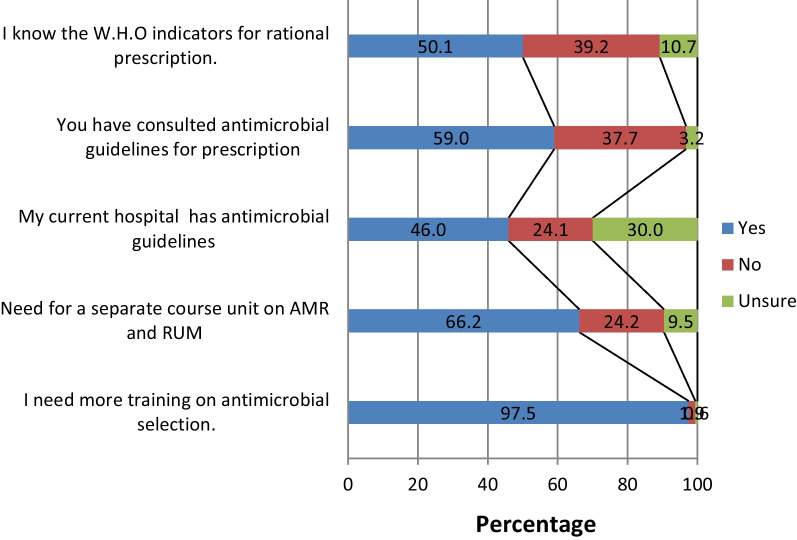
Perception of participants on their training on antimicrobial resistance and rational use of medicine

Over half of the participants (*n* = 371, 54.5%) agreed that antimicrobials were overused at their university teaching hospitals and that poor infection control practices by healthcare professionals causes spread of antimicrobial resistance (*n* = 318, 46.7%). About a third of the students (*n* = 277, 40.7%) thought that new antimicrobials will be developed in the future to keep up with the escalating problem of antimicrobial resistance (Table 4). Most participants (97.5%) agreed that they need more training on antimicrobial selection and a separate course unit on AMR and rational use of medicine (66.2%), (Fig. 3).

**Table 4:** Perceptions of participants towards antimicrobial resistance

Statement	SA (*n*, %)	A (*n*, %)	N (*n*, %)	D (*n*, %)	SD (*n*, %)
Prescribing broad-spectrum antimicrobials when equally effective narrower spectrum antimicrobials are available increases antimicrobial resistance	480 (70.5)	163(23.9)	16 (2.4)	14 (2.1)	8 (1.2)
Strong knowledge of antimicrobials is important in my medical career	520 (76.4)	155 (22.8)	6 (0.8)	0 (0)	0 (0)
Poor infection control practices by healthcare professionals cause spread of antimicrobial resistance	318 (46.7)	303 (44.5)	32 (4.7)	21 (3.1)	7 (1.0)
Excessive use of antimicrobials in livestock causes antimicrobial resistance	301 (44.2)	277 (40.7)	71 (10.4)	30 (4.4)	2 (0.3)
New antimicrobials will be developed in the future that will keep up with the problem of resistance	110 (16.2)	277 (40.7)	170 (24.9)	99 (14.5)	25 (3.7)
Antimicrobials are overused at the hospitals where I have rotated	196 (28.8)	371 (54.5)	91 (13.4)	22 (3.2)	1 (0.2)

*SA* strongly agree, *A* agree, *N* neutral, *D* disagree, *SD* strongly disagree

### Confidence level of participants on prescribing antimicrobials

Two thirds of the participants (*n* = 456, 66.9%), were confident on making an accurate diagnosis of infection/sepsis, choosing the correct antimicrobial to use (*n* = 484, 71.1%) and using a combination therapy if appropriate (*n* = 443, 65.1%), (Table 5). There was a statistically significant difference in confidence level scores among classes with highest scores among year 5 on making an accurate diagnosis of infection/sepsis (*p* < 0.001), choosing the correct antimicrobial to use (*p* = 0.019), choosing the correct dose and interval of administration (*p* = 0.019) among others.

**Table 5:** Confidence level of clinical year medical students to prescribe antimicrobials

Statement	VC (*n*, %)	C (*n*, %)	U (*n*, %)	UC (*n*, %)	VUC (*n*, %)
Making an accurate diagnosis of infection/sepsis	121(17.8)	456 (66.9)	24 (3.5)	54 (7.9)	26 (3.8)
Choosing the correct antimicrobial to use	72 (10.6)	484 (71.1)	24 (3.5)	82 (12.0)	19 (2.8)
Choosing the correct dose and interval of administration	86 (12.6)	439 (64.5)	26 (3.8)	108 (15.9)	22 (3.2)
Using a combination therapy if appropriate	67 (9.8)	443 (65.1)	31 (4.6)	115 (16.9)	25 (3.7)
Choosing between intravenous and oral administration	115 (16.9)	458 (67.2)	22 (3.2)	63 (9.3)	23 (3.4)
Planning to streamline/stop the antimicrobial treatment, according to clinical evaluation and investigations	66 (9.7)	425 (62.4)	63 (9.3)	106 (15.6)	21 (3.1)

*VC* very confident, *C* confident, *U* uncertain, *UC* unconfident, *VUC* very unconfident

## Discussion

The greatest increase in AMR and related deaths are estimated to occur in sub-Saharan Africa if nothing is done to avert the current trends [24]. This study explored the knowledge, confidence level of prescription, perceptions on antimicrobial resistance and training on antimicrobial use of health professions students in Uganda.

Our findings suggest that almost 9 in 10 participants had sufficient knowledge about AMR, especially the male students and those that had received prior additional training on AMR. This finding is in agreement with earlier findings around medical students in, Nigeria [25], Egypt [26], South Africa [27] and China [14, 28] which showed that medical students generally were more knowledgeable about antibiotics use. Contrary to this, previous findings among final year medical students in Uganda and Kenya report otherwise; only 36.6% (120/328) of students had good overall total knowledge [29]. The variations in the findings of these studies could be attributed to the fact that our study had a larger sample size i.e., 681 participants from 9 medical schools versus 328 participants in a study by Lubwama et al., 2021 from 3 medical Schools [29]. Furthermore, our study included students pursuing other medical courses i.e., Bachelor of Dental Surgery (BDS), Bachelor of Science in Nursing (BNS), and Bachelors of Anesthesia (BNA) which is not the case with the other study. Our findings are also contrary to previous studies in other parts of the world that reported limited knowledge amongst final year medical students; in Thailand [30] and Australian [31]. Such discrepancies could probably be due to the geographical reasons where diseases targeted by antibiotics may not be equally prevalent in the respectively settings. Also, the differences in medical curricula used, could also have an impact on the overall students’ knowledge regarding antibiotic use and antimicrobial resistance.

The knowledge scores of students on AMR increased up the years peaking at fifth year and a significant knowledge difference was observed among third years against fourth and fifth years. Also, the medicine students scored significantly higher than nursing students on AMR knowledge. Similar findings are drawn from other studies in Malaysia and China [2, 14]**.** As students’ progress in their respective academic programs, they get more encounters with antimicrobials, start prescribing and administering them under supervision thus garner more knowledge. Despite our study not showing a strong evidence on the effect of sex on AMR, males have been found to have poorer levels of knowledge compared to females [26].

Students in our study mainly used Medscape (47.7%), guidelines from professional bodies (31.0%), and UpToDate (30%) and as their sources of information on AMR. This is however in disagreement, with an earlier study among Chinese medical students which found textbooks or study guides, peers, and Wikipedia to be the topmost used resources of information [28]. There is need to ensure that medical students use the right information sources to help them acquire quality and updated good AMR knowledge. Over the past decades, there has been a significant increase in the number of educational websites and smartphone applications with information on drug prescriptions. Some of these include in addition to UpToDate, drugs.com, Medline, WebMD, Mayo Clinic, rxlist.com, goodrx.com, etc. Health professional students can utilize these readily available sources to improve their prescription practices.

It is encouraging to realize that most students in our study (over 80%) were aware about the essential medicines, and rational use of medicine, and could name prescription parts. This is a good indicator on their pharmacological training that could translate into better practices towards use of antimicrobials. The majority also appreciated the fact that antibiotics don’t speed up recovery of common cold because this is a great contributor to misuse of antibiotics. Over half of the students (54.5%) reported that antimicrobials were overused at their university teaching hospitals. This is consistent with several other Global Point Prevalence surveys done in hospitals in Uganda, Tanzania, Ghana, and other international reviews [32–34]. This shines a light on the pressing drivers of resistance that should be addressed within our hospitals by establishment of medicines and therapeutic committees, and/or infection prevention committees in which students’ representatives can be invited to become members.

About two-thirds of participants in our study believed that strong knowledge of antimicrobials is important in for their medical career. This result has also been previously documented among Chinese medical student by Yang et al.,2016[15]. The role of having strong antimicrobial knowledge can’t be overemphasized in antimicrobial stewardship, because it’s what drives antibiotics prescribing behaviors of health care workers [28]. There is need for more efforts to be directed towards improvement of medical students’ knowledge of antibiotics and their rational use of medicines, among especially those medical students with poor knowledge, attitudes and perception regarding AMR for example among Japanese medical students, of whom only 43 (6.5%) had ever heard about the AMR [35].

Unlike several systematic reviews that have consistently reported unpreparedness and low self-reported confidence of medical students to prescribe antimicrobials [21, 36, 37]**,** majority of our respondents were confident on choosing the correct antimicrobial to use (71.1%) and using a combination therapy if appropriate (65.1%). This could be due to for instance having 430 (89.4%) of students in our sample having had prior AMR training. Furthermore, the level of confidence was significantly highest among fifth year students possibly because they are the most senior and have encountered patients and prescriptions often. But this is also an encouraging finding as these confident students are soon entering into the health system. Thus, the confidence exhibited should be built on to create strong stewardship towards AMR.

Although students exhibited high confidence and good knowledge, almost all of them (97.5%) believed they need more training on antimicrobial selection and a separate course unit on AMR and rational use of medicine. This is in consonance with various other KAP studies on AMR among students [14, 19, 38] which underscores the need to rethink the medical curriculum in line with delivery of pharmacology course to put emphasis on antimicrobial prescription and resistance. The largest proportion of students knew about their teaching hospital antimicrobial guidelines and had consulted them.

### Limitations and strengths

This is a cross-sectional study that only provides a snippet of the prevailing situation at the time. The convenience sampling method used could have created bias in the different categories with few numbers represented in some however we tried to control for this by consistently sharing the tool across all groups. The use of WhatsApp as a data collection platform presents challenges of who accesses the data collection tool link that we could not completely control however we tried correcting for this by having focal persons per participating site to only share in specified groups with the targeted population. However, our study involved many health professions students across different universities and courses therefore the findings can be generalized with much confidence.

## Conclusion

This survey indicates that there is good knowledge among health profession students on antimicrobial resistance and rational use of medicine however they still demanded that a separated course on this is necessary. Responsible authorities should consider introducing this course unit in the curriculum of medical schools to consolidate this knowledge that can translate into good practices of antimicrobial use. The high self-perceived confidence level of students on prescription of antimicrobials should be leveraged to encourage to translate the exhibited good knowledge into better practice later when they start prescribing antimicrobials without supervision (Additional file 1).

## Supplementary Information

**Additional file1**. Study Questionnaire.

## Data Availability

The data collection tool used for this study is attached. All data generated or analyzed during the current study is not publicly available due to some individualized information it contains but are available from the corresponding author on reasonable request.

## Abbreviations

AMR: Antimicrobial resistance
WHO: World Health Organization
INRUD: International Network of Rational Use of Drugs
MBChB: Bachelor of Medicine and Bachelor of Surgery
BDS: Bachelor of Dental Surgery
BNS: Bachelor of Science in Nursing
BPharm: Bachelor of Pharmacy
BNA: Bachelors of Anesthesia
