# Supplementary material for: Antimicrobial resistance and rational use of medicine: knowledge, perceptions, and training of clinical health professions students in Uganda
Source: Antimicrob Resist Infect Control. 2022 Nov 25;11:145. doi: 10.1186/s13756-022-01186-9 (PMC9700951; doi:10.1186/s13756-022-01186-9)
Supplement: Supplementary file 1 — Additional file1. Study Questionnaire. [file 13756_2022_1186_MOESM1_ESM.docx]

# APPENDIX I: QUESTIONNAIRE

**Part A. Demographics**

1. Age …………
2. Sex
3. Male
4. Female
5. University
6. Makerere University
7. Busitema University
8. Islamic University in Uganda
9. Lira University
10. Mbarara University of Science and Technology
11. Gulu University
12. Kampala International University
13. Kabale University
14. Bishop Stuart University
15. Year Of Study
16. Year 3
17. Year 4
18. Year 5
19. Course
20. MBChB
21. BDS
22. BNA
23. BNS
24. BPharm
25. Do you have any added qualification?
26. Certificate (Nursing, Pharmacy, Midwifery, etc)
27. Diploma (Clinical Officer, Pharmacy, Nursing, etc)
28. Degree (BSB, BNUR, etc).
29. None
30. Have you had any prior training on rational prescription of drugs?

A. Yes

B. No

1. If yes above, where did you receive training from?
2. Medical school curriculum
3. Conference / Workshop
4. Other……………………….

**Part B: knowledge of antimicrobial resistance and rational use of medicine**

**Knowledge of Students on Antimicrobial resistance**

1. The national prevalence of Methicillin-resistant Staphylococcus aureus (MSRA) varies from 2% to 50%
2. Yes
3. No
4. Not sure
5. Resistance to first line anti-TB drug isoniazid in Uganda is approximately 5-20 percent resistance to isoniazid;
6. Yes
7. No
8. Not sure
9. Do antibiotics speed up the recovery of common cold and flu?
10. Yes
11. No
12. Not sure
13. Does the frequent use of antibiotics decrease its efficacy?
14. Yes
15. No
16. Not sure
17. Does inappropriate use of antibiotics put your patients at risk?
18. Yes
19. No
20. Not Sure

**Knowledge on rational use of medicine**

| No | Statement | Number of respondents | |
| --- | --- | --- | --- |
| 1. 17. | Are you aware of the term Rational use of medicine? | Yes | No |
| 1. 18. | Are you aware of the term essential medicines Lists (EML)? |  |  |
| 1. 19. | Are you aware of the term P-drugs? |  |  |
| 1. 20. | Can you name the parts of a prescription? |  |  |
| 1. 22. | Are you aware of STEP (Safety, tolerability, efficacy, price) criteria for selection of P-drug? |  |  |

1. Sources of Information on antimicrobial resistance and rational use of medicines
2. UpToDate
3. Medscape
4. Hospital Pharmacist
5. Non-infectious disease physicians
6. Infectious disease specialists
7. Medical journals
8. Peers (Other students)
9. Guidelines by professional organizations
10. Wikipedia
11. Pharmaceutical representatives
12. Others……………………

**Part C: Perceptions of students about Antimicrobial Resistance and training (Choose only one most appropriate option for you per row)**

**Perceptions of students about Antimicrobial Resistance**

| No. | Statement | Strongly Agree | Agree | Neutral | Disagree | Strongly Disagree |
| --- | --- | --- | --- | --- | --- | --- |
|  | Prescribing broad-spectrum antimicrobials when equally  effective narrower spectrum antimicrobials are available  increases antimicrobial resistance |  |  |  |  |  |
|  | Strong knowledge of antimicrobials is important in my  medical career |  |  |  |  |  |
|  | Poor infection control practices by healthcare professionals  cause spread of antimicrobial resistance |  |  |  |  |  |
|  | Excessive use of antimicrobials in livestock causes antimicrobial resistance |  |  |  |  |  |
|  | Antimicrobials are overused at the hospitals where I have  Rotated |  |  |  |  |  |
|  | New antimicrobials will be developed in the future that will  keep up with the problem of “resistance” |  |  |  |  |  |

**Perceptions on training of antimicrobial prescription**

| No. | Statement | Yes | No | Unsure |
| --- | --- | --- | --- | --- |
|  | More training on antimicrobial selection |  |  |  |
|  | Need for a separate course unit on antimicrobial resistance and rational use of medicines. |  |  |  |
|  | Current hospital has antimicrobial guidelines |  |  |  |
|  | You have used or consulted antimicrobial guidelines when considering an antimicrobial |  |  |  |
|  | You know about the W.H.O indicators for rational antimicrobial prescription |  |  |  |

**Part D: Confidence level of antimicrobial prescription among medical Students**

| No. | QUESTION | Very unconfident | Unconfident | Confident | Very Confident | Uncertain |
| --- | --- | --- | --- | --- | --- | --- |
|  | Making an accurate diagnosis of infection/sepsis |  |  |  |  |  |
|  | Choosing the correct antimicrobial to use |  |  |  |  |  |
|  | Choosing the correct dose and interval of administration |  |  |  |  |  |
|  | Using a combination therapy if appropriate |  |  |  |  |  |
|  | Choosing between intravenous and oral administration |  |  |  |  |  |
|  | Planning to streamline/stop the antimicrobial treatment, according to clinical evaluation and investigations |  |  |  |  |  |
